# SRC kinase inhibition with saracatinib limits the development of osteolytic bone disease in multiple myeloma

**DOI:** 10.18632/oncotarget.8750

**Published:** 2016-04-15

**Authors:** Roy Heusschen, Joséphine Muller, Marilène Binsfeld, Caroline Marty, Erwan Plougonven, Sophie Dubois, Nadia Mahli, Karen Moermans, Geert Carmeliet, Angélique Léonard, Frédéric Baron, Yves Beguin, Eline Menu, Martine Cohen-Solal, Jo Caers

**Affiliations:** ^1^ Laboratory of Hematology, GIGA-Research, University of Liège, Liège, Belgium; ^2^ Division of Hematology, Department of Medicine, University and CHU of Liège, Liège, Belgium; ^3^ INSERM-UMR-1132, Hôpital Lariboisière and Université Paris Diderot, Paris, France; ^4^ Department of Chemical Engineering, PEPs (Products, Environments, Processes), University of Liège, Liège, Belgium; ^5^ Laboratory of Clinical and Experimental Endocrinology, Department of Clinical and Experimental Medicine, KU Leuven, Leuven, Belgium; ^6^ Department of Hematology and Immunology, Myeloma Center Brussels, Vrije Universiteit Brussel, Brussels, Belgium

**Keywords:** multiple myeloma, osteolytic bone disease, c-SRC, saracatinib, osteoclast

## Abstract

Multiple myeloma (MM)-associated osteolytic bone disease is a major cause of morbidity and mortality in MM patients and the development of new therapeutic strategies is of great interest. The proto-oncogene SRC is an attractive target for such a strategy. In the current study, we investigated the effect of treatment with the SRC inhibitor saracatinib (AZD0530) on osteoclast and osteoblast differentiation and function, and on the development of MM and its associated bone disease in the 5TGM.1 and 5T2MM murine MM models. *In vitro* data showed an inhibitory effect of saracatinib on osteoclast differentiation, polarization and resorptive function. In osteoblasts, collagen deposition and matrix mineralization were affected by saracatinib. MM cell proliferation and tumor burden remained unaltered following saracatinib treatment and we could not detect any synergistic effects with drugs that are part of standard care in MM. We observed a marked reduction of bone loss after treatment of MM-bearing mice with saracatinib as reflected by a restoration of trabecular bone parameters to levels observed in naive control mice. Histomorphometric analyses support that this occurs through an inhibition of bone resorption. In conclusion, these data further establish SRC inhibition as a promising therapeutic approach for the treatment of MM-associated osteolytic bone disease.

## INTRODUCTION

Multiple myeloma (MM) is the second most common hematological malignancy and accounts for approximately 1% of all cancers and 2% of all deaths from cancer. In the past decades, therapeutic advances have been made by introducing hematopoietic stem cell transplantation and new targeted molecules such as immune-modulatory agents, proteasome inhibitors and monoclonal antibodies. Unfortunately, MM remains an incurable disease with a 10-year survival of approximately 30% in patients under 60 years [1]. MM is characterized by the clonal proliferation and accumulation of malignant plasma cells in the bone marrow (BM), monoclonal serum protein and associated organ dysfunction [2]. Underlying these changes are the oncogenic transformation of plasma cells and an associated altered BM microenvironment that further supports MM development [3].

A major cause of morbidity and mortality in MM is the development of bone destructive lesions due to osteolytic bone disease, which occurs in more than 80% of MM patients. These lesions can be extensive and cause severe bone pain, hypercalcemia, spinal cord compression and pathologic fractures that require radiation or surgical intervention. It is estimated that 50% of patients develop pathologic fractures over the course of their disease, increasing the risk of death by more than 20% compared to patients without fractures [4, 5]. MM-associated osteolytic bone disease is characterized by increased osteoclastogenesis and suppression of osteoblast function. This occurs via multiple mechanisms, including the secretion of osteoclastogenic factors and osteoblast-inhibitory factors by both MM cells and stromal cells in the MM microenvironment. As a result, the bone remodeling process is uncoupled culminating in bone loss and lytic lesions [6–8].

The proto-oncogene *c-SRC* (*SRC*), a member of the SRC family of protein tyrosine kinases (SFKs), is a non-receptor tyrosine kinase that mediates signal transduction from a diverse set of cell surface receptors in a wide range of cellular processes, including proliferation, differentiation, motility, adhesion and survival [9, 10]. SRC has been suggested to play an equally important role in osteoclasts and osteoblasts. SRC levels increase during osteoclast differentiation and SRC is important for the formation of actin rings in mature osteoclasts [11, 12]. Contrary to osteoclasts, SRC levels appear to decrease during osteoblast differentiation [13, 14]. As a result, depletion of SRC expression enhances osteoblast differentiation and bone formation [15]. These findings correspond with the osteopetrotic phenotype observed in *SRC−/−* mice [16], which display hepatosplenomegaly and develop odontomas with age [17].

As described, MM-associated osteolytic bone disease not only has a negative impact on the quality of life but also results in morbidity and adversely impacts overall survival of MM patients. Bisphosphonates remain the standard of care for MM-associated osteolytic bone disease and slow the progression of osteolytic lesions, prevent the development of pathologic fractures and may have additional limited anti-tumor effects in MM [18]. Interestingly, recent reports suggest that bisphosphonates act in part by inhibiting *SRC* expression [19] or modulating SRC signaling [20]. However, bisphosphonate use can have adverse side effects such as renal impairment, the development of atypical fractures and avascular necrosis of the jaw. Similar adverse effects were observed with novel targeted drugs such as the monoclonal receptor activator of nuclear factor κB ligand (RANKL)-antibody denosumab [21]. Moreover, skeletal-related events still occur in approximately 25% of patients receiving bisphosphonate therapy [22]. Thus, the development of new therapeutic strategies for this MM-related bone disease is of great interest. SRC is a promising target for such a strategy, given its important role in osteoclast and osteoblast function. Saracatinib is an orally available ATP-competitive SRC inhibitor which has been shown to hamper osteoclast function. In the current study, we investigated the effect of saracatinib on osteoclast and osteoblast function, and on the development of MM and its associated osteolytic bone disease.

## RESULTS

### Expression of SRC family kinases in the multiple myeloma microenvironment

**Figure 1:**
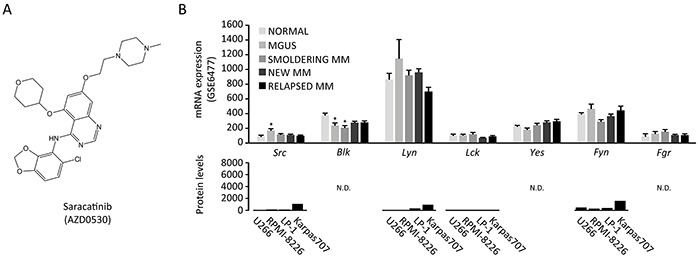
Expression of SRC family kinases in multiple myeloma cells **A.** Chemical structure of saracatinib (4-Quinazolinamine, N-(5-chloro-1,3-benzodioxol-4-yl)-7-[2-(4-methyl-1-piperazin-1-yl)ethoxy]-5-(tetrahydro-2H-pyran-4-yloxy)-quinazolin-4-amine) (image source: selleckchem.com). **B.** SFK mRNA expression in patients (GSE6477 dataset, total n=162 patients): Normal (healthy controls, n=15), MGUS (monoclonal gammopathy of undetermined significance, n=21), smoldering MM (n=23), newly diagnosed MM (n=75) and relapsed MM (n=28)(upper panel, *: p<.0.05 versus normal). SFK protein levels in MM cell lines (proteinatlas.org, lower panel). N.D.: not determined. Y-axis is kept at 0-8000 to illustrate the protein expression range in cell types of different origin in the proteinatlas.org database.

Saracatinib (Figure 1A) is a potent SRC inhibitor with an IC50 of 2.7 nM in cell-free assays [23]. Aside SRC, other SFKs are potentially targeted by this compound, including Lymphocyte Cell-Specific Protein-Tyrosine Kinase (LCK, 4 nM), Yamaguchi Sarcoma Oncogene (c-YES, 4 nM), Lck/Yes-Related Novel Protein Tyrosine Kinase (LYN, 5 nM), FYN Proto-Oncogene, SFK (FYN, 10 nM), Feline Gardner-Rasheed Sarcoma Viral Oncogene Homolog (FGR, 10 nM) and B Lymphoid Tyrosine Kinase (BLK, 11 nM). We assessed the expression of these SFKs in MM cells. We first determined their expression in MM cells in a large cohort of MM patients (n = 162) at different stages of the disease. Except *Lyn*, all SFKs are expressed at low levels in MM cells (Figure 1B). Barring a few exceptions, SFKs mRNA expression generally does not change during MM progression. These findings were corroborated by analysis of SFK protein levels in different MM cell lines, i.e. U266, RPMI-8226, LP-1 and Karpas707. If available, these data show very low to absent protein expression of SFKs in MM cell lines compared to cells of different origin (Figure 1B). We also performed SFKs expression analysis on publically available microarray data of osteoclast and osteoblast differentiation. We observed a marked increase in *Src* expression during osteoclast differentiation, while *Fgr* expression levels moderately increased. Other SFKs were expressed at very low levels (Supplementary. Figure S1A). During the differentiation from mesenchymal stem cells to fully differentiated osteoblasts, *Fyn* expression levels slightly increased. Conversely, *Src* mRNA levels decreased at day 14, followed by a return to initial levels in fully matured osteoblasts at day 21. Osteoblasts also expressed *Yes* and *Lyn* (Supplementary. Figure S1B).

### Saracatinib inhibits RAW264.7 and primary osteoclast differentiation and bone resorption

**Figure 2:**
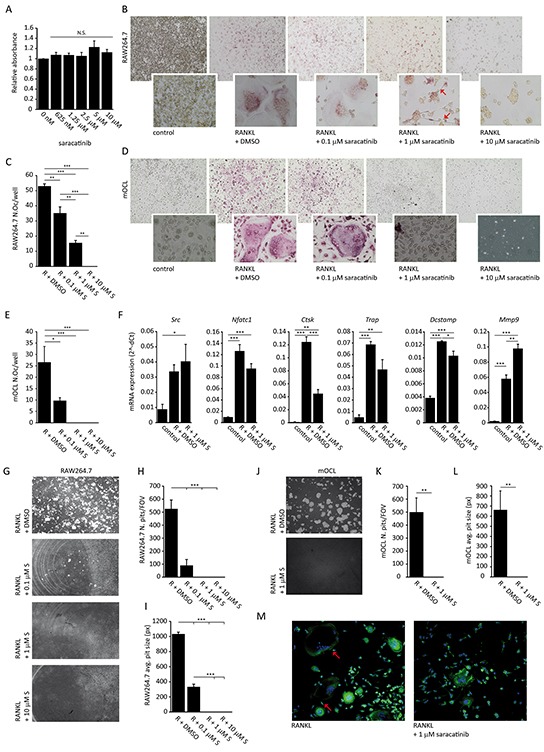
Effect of saracatinib on osteoclast differentiation and function **A.** MTT assay of RAW264.7 cells incubated with a range of saracatinib concentrations (n=6 experiments). N.S.: not significant compared to controls. **B.** Representative images of TRAP-stained RAW264.7 osteoclast cultures incubated with a range of saracatinib concentrations. Red arrows indicate pseudopodia (40x magnification, inserts 400x). **C.** Quantification of RAW264.7 osteoclast numbers per well (N.Oc/well) following RANKL (R) stimulation and incubation with saracatinib (S) (n=3 experiments, *: p<.0.05, **: p<0.01, ***: p<0.001). **D.** Representative images of TRAP-stained primary murine osteoclast (mOCL) cultures incubated with a range of saracatinib concentrations (40x magnification, inserts 400x). **E.** Quantification of primary mOCL numbers per well (N.Oc/well) following RANKL (R) stimulation and incubation with saracatinib (S) (n=4 experiments, *: p<.0.05, ***: p<0.001). **F.** mRNA expression levels of RAW264.7 osteoclast markers determined by qPCR. Controls represent cultures not stimulated with any cytokine. (n=3 experiments, *: p<.0.05, **: p<0.01, ***: p<0.001). **G.** Representative images of resorption pits generated by RAW264.7 osteoclasts incubated with a range of saracatinib concentrations (40x magnification). **H.** Quantification of the number of resorption pits per field of view (N. pits/FOV) (n=3 experiments, ***: p<0.001) and **I.** average pit size (n=3 experiments, ***: p<0.001) in RAW264.7 osteoclast cultures. **J.** Representative images of resorption pits generated by primary mOCLs treated with DMSO or 1 μM saracatinib (40x magnification). **K.** Quantification of the number of resorption pits per field of view (N. pits/FOV) (n=3 experiments, **: p<0.01) and (L) average pit size (n=3 experiments, **: p<0.01) in primary mOCL cultures. **M.** Representative confocal microscopy images of phalloidin-FITC staining of actin in RAW264.7 osteoclast cultures. Red arrows indicate actin rings in polarized osteoclasts. Blue: DAPI nuclear stain (200x magnification). All data are represented as mean +/− standard error.

Saracatinib treatment has previously been linked to decreased osteoclastogenesis and function. Here, we elaborated on and confirmed these findings. We first assessed whether saracatinib influences RAW264.7 proliferation and found no such effect over a range of concentrations after 3 days of culture (Figure 2A). Next, we confirmed an inhibitory effect of saracatinib on osteoclast generation by TRAP staining. We observed a concentration-dependent decrease in the number of osteoclasts, i.e. TRAP-positive cells with at least 3 nuclei, which already became apparent at 0.1 μM (Figure 2B and 2C). These findings were confirmed on primary murine osteoclasts (Figure 2D and 2E). Although lower concentrations of saracatinib did not affect cell survival, 10 μM appeared toxic in RANKL-stimulated cultures. *Src*, *Nfatc1* and *Trap* expression levels were not affected by saracatinib (Figure 2F). Conversely, saracatinib treatment resulted in a decreased expression of *Ctsk* and *Dc-stamp* and an increased expression of *Mmp-9* compared to DMSO-treated osteoclast cultures (Figure 2F). These changes culminated in defective matrix resorption by osteoclasts following saracatinib treatment (Figure 2G), as reflected in the total number of resorption pits (Figure 2H) and the average resorption pit size (Figure 2I). The inhibition of matrix resorption was already apparent after 0.1 μM saracatinib treatment, even though osteoclasts displayed a normal morphology at this concentration, and a complete inhibition of resorption was observed at concentrations > 1 μM. This inhibitory effect on matrix resorption was also confirmed on primary murine osteoclasts (Figure 2J, 5K and 2L). Finally, we found that actin ring formation is blocked after treatment with 1 μM saracatinib (Figure 2M).

### Saracatinib decreases collagen deposition and affects matrix mineralization by osteoblasts

**Figure 3:**
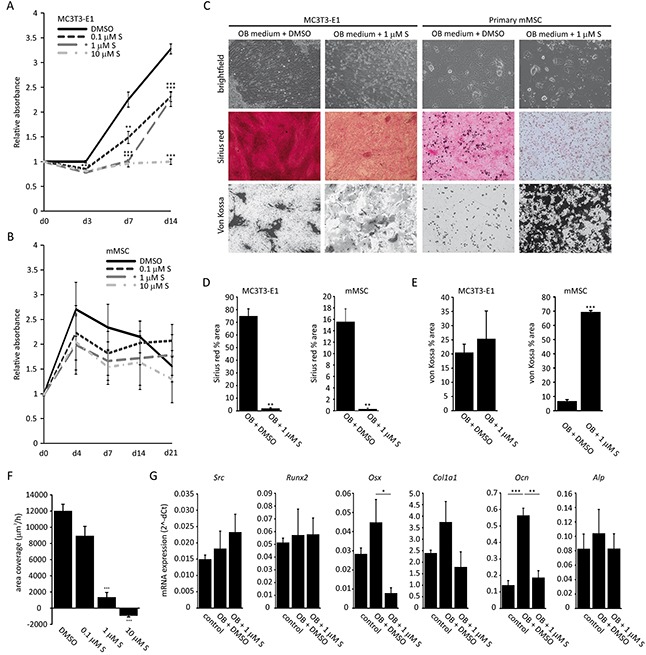
Effect of saracatinib on osteoblast function **A.** MTT assay on MC3T3-E1 cells incubated with a range of saracatinib concentrations (n=3 experiments, **: p<0.01, ***:p<0.001 compared to DMSO-treated control cultures). **B.** MTT assay on primary murine MSCs (mMSC) incubated with a range of saracatinib concentrations (n=3 experiments, no significant differences compared to DMSO-treated control cultures). **C.** Representative brightfield (200x magnification), Sirius red staining and von Kossa staining images (40x magnification) on MC3T3-E1 and mMSCs cultured in osteoblast differentiation medium (OB medium) with or without saracatinib (S). **D.** Quantification of Sirius red staining (percentage area) in osteoblast cultures (n=3 experiments, **: p<0.01). **E.** Quantification of von Kossa staining (percentage area) in osteoblast cultures (n=3 experiments, ***: p<0.001). **F.** Migration rates of MC3T3-E1 cells treated with a saracatinib (S) (n=3 experiments, ***: p<0.001). **G.** mRNA expression levels of MC3T3-E1 osteoblast markers determined by qPCR. Controls represent cultures in standard medium. (n=3 experiments, *: p<.0.05, **: p<0.01, ***: p<0.001). All data are represented as mean +/− standard error.

Whether saracatinib has a direct effect on osteoblast function has not yet been explored. We addressed this *in vitro* on the murine calvarial MC3T3-E1 cell line, a suitable model to study osteoblast differentiation and function [24], and on primary murine mesenchymal stem cell (MSC)-derived osteoblast cultures. We found that proliferation of MC3T3-E1 cells is hampered when treated with pharmacologically relevant saracatinib concentrations, i.e. a decrease of 31% relative absorbance on day 14 with a concentration of 1 μM saracatinib or lower versus controls (Figure 3A). No such an effect could be observed on primary MSCs (Figure 3B). Next, we investigated the effect of 1 μM saracatinib on osteoblast collagen deposition and matrix mineralization by Sirius red staining and Von Kossa staining, respectively (Figure 3C). Osteoblast size appeared increased in saracatinib-treated cultures and coincided with drastically decreased collagen deposition (Figure 3D). The total degree of matrix mineralization remained unaltered in MC3T3-E1 osteoblast cultures (Figure 3E) although the pattern of mineralization appeared different in saracatinib-treated cultures with less discerned nodules. Conversely, matrix mineralization markedly increased in MSC osteoblast cultures (Figure 3E). We found a dose dependent inhibitory effect of saracatinib on MC3T3-E1 migration (Figure 3F). Finally, we assessed the effect of saracatinib on osteoblast marker gene expression in MC3T3-E1 cells and observed a decrease of *Osx* and *Ocn* expression. In addition, *Col1a1* expression was diminished, although this did not reach statistical significance. Saracatinib treatment did not alter *Src*, *Runx2* or *Alp* expression (Figure 3G).

### Saracatinib does not influence myeloma cell proliferation or bone marrow plasmocytosis in murine multiple myeloma models

**Figure 4:**
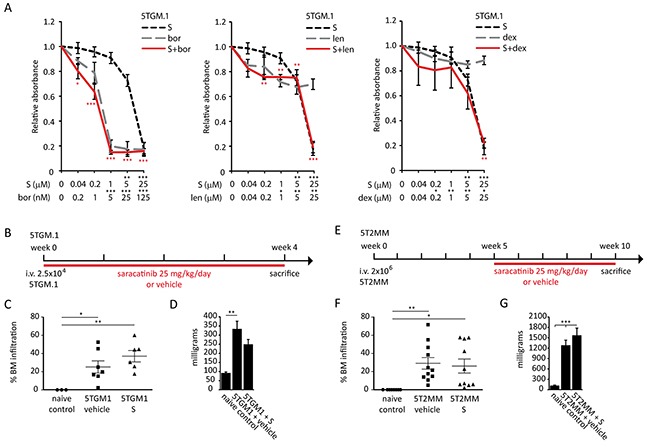
Effect of saracatinib, and combinations with standard drugs, on multiple myeloma cell proliferation, and bone marrow plasmocytosis **A.** MTT assays on 5TGM.1 myeloma cells treated with saracatinib (S), bortezomib (bor), lenalidomide (len), dexamethasone (dex) or combinations of saracatinib with these drugs. Drug concentrations and significance levels of single drug treatments compared to controls are noted on the X-axis. Significance of drug combinations compared to controls are noted near the data points in the figure (in red). No significant synergistic or additive effects were detected (n=3 experiments, *: p<.0.05, **: p<0.01, ***: p<0.001). **B.** Saracatinib treatment schedule in the 5TGM.1 cohort. **C.** Percentage BM infiltration of MM cells in naive controls, vehicle- and saracatinib (S)-treated mice in the 5TGM.1 cohort (n=3/7/6 bones, *: p<.0.05, **: p<0.01) as determined by FACS. **D.** Spleen weight as measured at the time of sacrifice in the 5TGM.1 cohort (n=3/7/6 spleens, **: p<0.01). **E.** Saracatinib treatment schedule in the 5T2MM cohort. **F.** Percentage BM infiltration of MM cells in naive controls, vehicle- and saracatinib (S)-treated mice in the 5T2MM cohort (n=8/11/10 bones, *: p<.0.05, **: p<0.01) as determined on cytosmears. **G.** Spleen weight as measured at the time of sacrifice in the 5T2MM cohort (n=8/11/10 spleens, ***: p<0.001). All data are represented as mean +/− standard error.

We first determined the effect of saracatinib on the proliferation of MM cell lines. We found that saracatinib only hampers murine 5TGM.1 MM cell proliferation at concentrations of 5 μM or higher (Figure 4A). Similar effects were observed on human U266, JJN-3 and LP-1 myeloma cell lines and murine 5T33 cell proliferation was not affected by saracatinib (Supplementary. Figure S2). Thus, saracatinib has no effect on MM cell proliferation at the pharmacologically relevant saracatinib concentration of 1 μM or lower. In addition, we performed combination studies in which saracatinib was combined with drugs that are part of standard care for MM, i.e. bortezomib, lenalidomide and dexamethasone. Similarly, we could observe no additive or synergistic effects when combining saracatinib with these drugs (Figure 4A and Supplementary. Figure S2). Next, we assessed the efficacy of saracatinib to hamper MM development *in vivo* in 2 different murine models. In the 5TGM.1 model, daily treatment was initiated one day after injection of 5TGM.1 cells and continued during the course of the disease (Figure 4B). BM infiltration by MM cells in saracatinib-treated mice (37+-6.28%) was not different from that in vehicle-treated mice (25.24+-6.67%) (Figure 4C). In addition, there was no difference in spleen weight between saracatinib- and vehicle-treated mice (Figure 4D). In the 5T2MM model, daily treatment was initiated 5 weeks after injection of 5T2MM cells (Figure 4E). At this moment, a monoclonal paraprotein could be detected in the serum of diseased mice and the treatment schedule corresponds to the clinical situation where treatment is started upon diagnosis. Similar to the findings in the 5TGM.1 model, we observed no effect on BM infiltration by MM cells (26.10+-7.71% vs. 29.08+-6.35%) (Figure 4F) or on spleen weight (Figure 4G). In conclusion, saracatinib did not hamper the proliferation of MM cells in the BM. Of note, we observed an almost complete absence of any sign of paraplegia, caused by spinal cord compression by tumor masses or vertebral collapse, in saracatinib-treated mice in the 5TGM.1 model at the moment of sacrifice (data not shown), suggesting improved bone strength in these mice.

### Saracatinib treatment limits the development of osteolytic bone disease in the 5TGM.1 and 5T2MM multiple myeloma models

**Figure 5:**
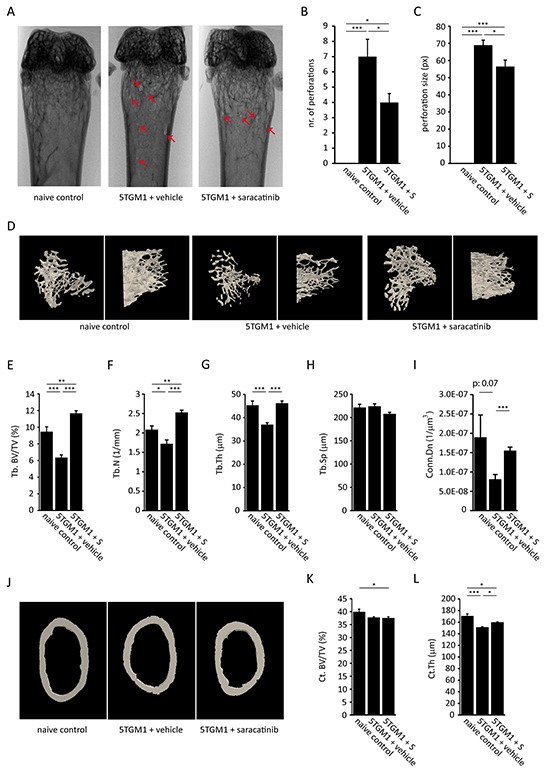
Effect of saracatinib treatment on the development of bone disease in the 5TGM.1 multiple myeloma model **A.** Representative distal femur radiographs of naive control mice, vehicle-treated myeloma-bearing mice and saracatinib (S)-treated myeloma-bearing mice. Red arrows indicate cortical perforations. **B.** Quantification of the number of cortical perforations (n=6/7/7 bones, *: p<.0.05, ***: p<0.001). **C.** Quantification of perforation size (n=6/7/7 bones, *: p<.0.05, ***: p<0.001). **D.** Representative reconstructed 3D-models of trabecular bone of the proximal tibia in naive control mice, vehicle-treated mice and saracatinib-treated mice. Left panels show a top-down view, right panels show a lateral view. **E.** Trabecular bone volume (Tb.BV/TV), **F.** trabecular number (Tb.N), **G.** trabecular thickness (Tb.Th), **H.** trabecular separation (Tb.Sp) and **I.** connectivity density (Conn.Dn) as determined by μCT morphometric analysis on proximal tibiae (n=6/7/7 bones, *: p<.0.05, **: p<0.01, ***: p<0.001). **J.** Representative reconstructed 3D-models of cortical bone of the distal femur in naive control mice, vehicle-treated mice and saracatinib-treated mice. **K.** Cortical bone volume (Ct. Bv/TV) and **L.** cortical thickness (Ct.Th) as determined by μCT morphometric analysis on distal femurs (n=6/7/7 bones, *: p<.0.05, ***: p<0.001). All data are represented as mean +/− standard error.

**Figure 6:**
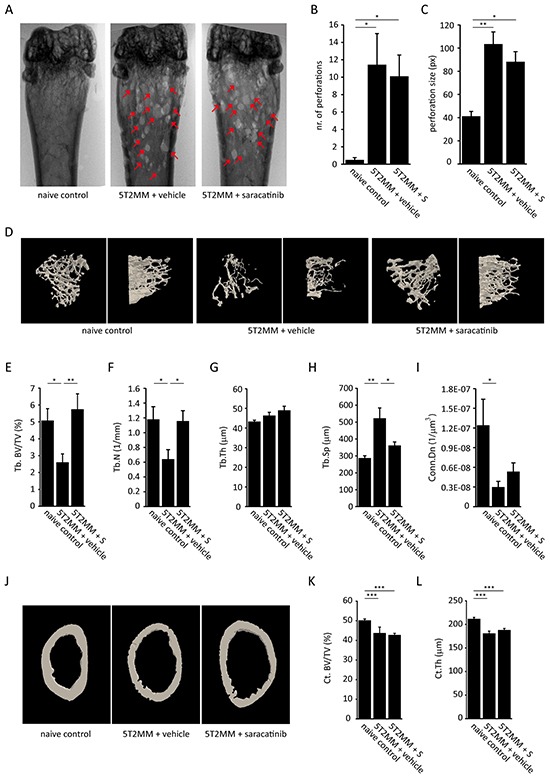
Effect of saracatinib treatment on the development of bone disease in the 5T2MM multiple myeloma model **A.** Representative distal femur radiographs of naive control mice, vehicle-treated myeloma-bearing mice and saracatinib (S)-treated myeloma-bearing mice. Red arrows indicate cortical perforations. **B.** Quantification of the number of cortical perforations (n=8/12/10 bones, *: p<.0.05). **C.** Quantification of perforation size (n=8/12/10 bones, *: p<.0.05, **: p<0.01). **D.** Representative reconstructed 3D-models of trabecular bone of the proximal tibia in naive control mice, vehicle-treated mice and saracatinib-treated mice. Left panels show a top-down view, right panels show a lateral view. **E.** Trabecular bone volume (Tb.BV/TV), **F.** trabecular number (Tb.N), **G.** trabecular thickness (Tb.Th), **H.** trabecular separation (Tb.Sp) and **I.** connectivity density (Conn.Dn) as determined by μCT morphometric analysis on proximal tibiae (n=8/12/10 bones, *: p<.0.05, **: p<0.01). **J.** Representative reconstructed 3D-models of cortical bone of the distal femur in naive control mice, vehicle-treated mice and saracatinib-treated mice. **K.** Cortical bone volume (Ct. Bv/TV) and **L.** cortical thickness (Ct.Th) as determined by μCT morphometric analysis on distal femurs (n=8/12/10 bones, ***: p<0.001). All data are represented as mean +/− standard error.

The effect of saracatinib treatment on trabecular and cortical bone parameters in myeloma-bearing mice was assessed by μCT. We tested saracatinib in both the 5TGM.1 model and the 5T2MM model. Cortical perforations were readily visible on radiographs (Figure 5A and 6A).

In the 5TGM.1 model, saracatinib treatment resulted in a decrease in the number (Figure 5B) and size (Figure 5C) of cortical perforations. Although perforations were observed in every portion of the bone, they predominantly occurred at the metaphyses. Next, we assessed trabecular bone parameters (Figure 5D) and found that trabecular bone volume (Tb. BV/TV) (Figure 5E) was not only restored, but exceeded that of naïve control mice. This was due to a restoration of trabecular number (Tb.N) (Figure 5F) and trabecular thickness (Tb.Th) (Figure 5G) to levels observed in naive control mice following saracatinib treatment. In addition, connectivity density (Conn.Dn) (Figure 5I) values were restored. Conversely, there was no significant effect on trabecular separation (Tb.Sp) (Figure 5H). As for cortical bone parameters, we found no effect of saracatinib on cortical bone volume (Ct. BV/TV) (Figure 5J and 5K). Although cortical thickness (Ct.Th) was slightly restored by saracatinib treatment (Figure 5L), it did not reach the level of that observed in healthy mice. The marrow area, endosteal and periosteal perimeter were lower in MM bearing mice and saracatinib treatment did not affect these parameters (Supplementary. Figure S3A).

In the 5T2MM model there was no effect of saracatinib treatment on the number (Figure 6B) or size (Figure 6C) of cortical perforations and, similar to the 5TGM.1, these perforations occurred predominantly at the metaphyses. However, even though the osteolytic bone disease is more severe in this model (Figure 6D), we observed a complete restoration of Tb. BV/TV (Figure 6E) in the saracatinib-treated group compared to vehicle-treated controls. In this model, the restoration of Tb. BV/TV was due to an increase in Tb.N (Figure 6F) and not Tb.Th (Figure 6G). Tb.Sp (Figure 6H) was restored and Conn.Dn (Figure 6I) was not affected by saracatinib treatment. Similar to the occurrence of cortical perforations, we found no effect of saracatinib treatment on Ct. BV/TV (Figure 6J and 6K) or Ct.Th (Figure 6L) in this model. Similar to the 5TGM.1 model, we observed no differences in porosity, marrow area, periosteal and endosteal perimeter between vehicle- and saracatinib-treated myeloma-bearing mice (Supplementary. Figure S3B).

**Figure 7:**
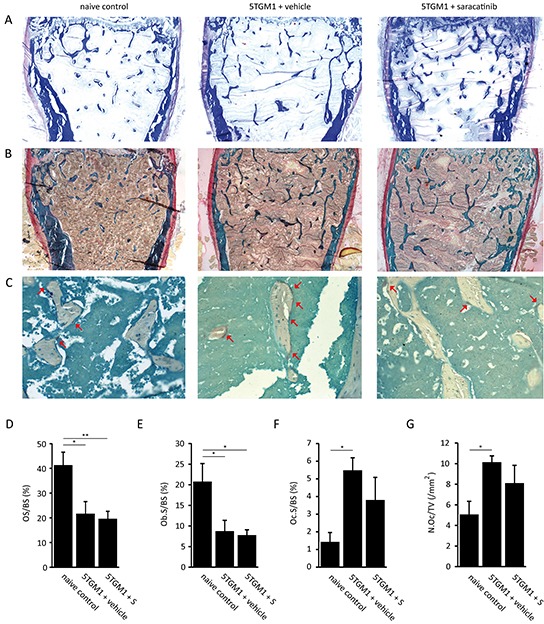
Histomorphometric analysis of bone parameters in the 5TGM.1 multiple myeloma model **A.** Representative images of aniline blue stained bone sections from naive control mice, vehicle-treated myeloma-bearing mice and saracatinib (S)-treated myeloma-bearing mice (n=5/7/7 bones) (40x magnification). **B.** Representative images of Goldner's trichrome stained bone sections from naive control mice, vehicle-treated mice and saracatinib-treated mice (n=5/7/7 bones) (40x magnification). **C.** Representative images of bone sections from naive control mice, vehicle-treated mice and saracatinib-treated mice stained for TRAP-positive cells. Red arrows indicate osteoclasts (n=3/5/4 bones) (200x magnification). **D.** Osteoid surface (OS/BS), **E.** osteoblast surface (Ob.S/BS), **F.** osteoclast surface (Oc.S/BS) and **G.** osteoclast number (N.Oc/TV) were measured on these sections using the Bonolab software package (*: p<.0.05, **: p<0.01). All data are represented as mean +/− standard error.

Finally, histomorphometric analyses were performed on femurs from mice from the 5TGM.1 cohort. Toluidine blue, aniline blue (Figure 7A), Goldner's trichrome (Figure 7B) and TRAP stainings (Figure 7C) were performed to assess osteoid surface (OS/BS) and osteoblast surface (Ob.S/BS), and osteoclast surface (Oc.S/BS) and osteoclast number (Oc.N/TV) respectively. As expected, myeloma-bearing mice showed a decrease in OS/BS (Figure 7D) and Ob.S/BS (Figure 7E) compared to naive controls. Consistent with our *in vitro* data, saracatinib treatment did not result in a restoration of either of these parameters. Conversely, osteoclast surface (Figure 7F) and number (Figure 7G) were increased in myeloma-bearing mice. Although this did not reach statistical significance, saracatinib treatment resulted in a trend towards a decrease in both parameters.

## DISCUSSION

Even though considerable therapeutic advances have been made in recent years, MM remains an incurable hematological malignancy associated with high morbidity and mortality related to osteolytic bone disease. The characteristic bone loss and lytic lesions are caused by increased osteoclastogenesis and impaired osteoblast function. The finding that SRC plays an important role in osteoclast biology and has been implicated in osteoblast differentiation makes SRC a promising target for the treatment of MM-associated osteolytic bone disease.

*SRC* is constitutively expressed at high levels only in cells that are specialized for regulated secretion, i.e. neuronal cells, platelets and osteoclasts [10, 25]. Also, SRC is predominantly maintained in an inactive state in normal cells. Conversely, SRC levels or activity are increased in many types of cancer and SRC modulates several aspects of tumorigenesis, including tumor cell adhesion, migration, motility and survival [26]. This spurred the development of a wide range of SRC inhibitors, several of which are currently in clinical trials or are already approved for cancer therapy [26, 27]. Saracatinib is an orally available ATP-competitive SRC (IC50: 2.7 nM) inhibitor that also potently inhibits other SFKs members (IC50: 4-11nM) and ABL (IC50: 30nM) [23]. Although less potent in cell-free assays than inhibitors such as dasatinib, saracatinib has a higher selectivity for SRC and it is the first inhibitor to show inhibition of the SRC signaling pathway in human tumor tissue [28]. Moreover, initial studies indicate saracatinib is generally well-tolerated [29–31].

Osteoclast differentiation as well as their activity is increased in the MM microenvironment. This is mediated by cytokines, e.g. RANKL and macrophage colony-stimulating factor (M-CSF), which are secreted both by MM and stromal cells [6, 32]. SRC levels are high in mature osteoclasts and SRC mediates signaling downstream from α_v_β_3_ integrin, resulting in osteoclast polarization, RANK and the M-CSF receptor c-fms [33, 34]. De Vries *et al.* demonstrated that saracatinib reduces phosphorylated SRC levels in human osteoclast cultures, resulting in a reduced osteoclast differentiation and function, although without such an effect on PBMC-derived osteoclasts in monoculture [35]. We confirmed several of these findings in murine primary and RAW264.7 osteoclast cultures and expanded on them (Figure 2). Saracatinib did not hamper the proliferation or survival of RAW264.7 cells. In accordance with previous reports [35–37], we observed an inhibition of osteoclast differentiation, polarization and matrix resorption following SRC inhibition, which corresponded with a decrease in cathepsin K (*Ctsk*) levels. Of note, even 0.1 μM saracatinib almost completely blocked matrix resorption. Also, saracatinib induced a decrease in dendrocyte expressed seven transmembrane protein (*DC-Stamp*) expression and an increase in matrix-metalloproteinase 9 (*Mmp9*) expression, suggesting these osteoclasts are stuck in a pre-fusion state, with the presence of many pseudopodia, since DC-STAMP mediates osteoclast progenitor fusion [38] and, although not yet studied in the context of osteoclastogenesis, MMP9 has been implicated in the migration of monocytes [39, 40] and accumulates at the leading edge of pseudopodia [41, 42]. Defective osteoclast function after saracatinib treatment is in agreement with findings in phase I trials. Hannon *et al.* reported a decrease in bone resorption markers in healthy men and in patients with diverse advanced malignancies after saracatinib treatment [29, 30]. In addition, osteoclast function is impaired in *Src*−/− mice [16], which display an osteopetrotic phenotype with an inability of osteoclasts to form ruffled borders [43].

Osteoblast differentiation is suppressed in the MM microenvironment [6] and SRC protein levels have previously been shown to decrease during osteoblast differentiation [13, 14]. Here, we detected no differences in *Src* mRNA expression in mature osteoblasts compared to progenitor cells. Whether this is due to a different source of the cells or reflects a translational regulation of SRC in osteoblasts remains to be studied. Decreased *Src* expression inhibits immature osteoblast proliferation but enhances osteoblast differentiation, with a corresponding increase in nodule mineralization [15], which was also observed following dasatinib treatment of MC3T3-E1 derived osteoblasts [44]. We observed a strong decrease in collagen deposition following treatment of murine primary and MC3T3-E1 osteoblasts with 1 μM saracatinib, which could not be solely attributed to a decrease in osteoblast numbers (Figure 3). Similarly, matrix mineralization pattern or amount was affected by saracatinib. Interestingly, a similar large increase in von Kossa staining intensity was observed previously in SRC-knockdown or -deficient primary osteoblasts [15, 44]. Finally, we found saracatinib dose-dependently inhibits MC3T3-E1 migration, which corresponds with an inverse correlation between osteoblast migration and differentiation [45, 46]. It is currently not clear by which mechanism SRC inhibition leads to these effects and further studies are needed to address this. Peruzzi *et al.* have shown that SRC is involved in a regulatory loop together with IL-6 and insulin-like growth factor 5 (IGFBP5) maintaining osteoblasts in an immature state [14]. Multiple studies have reported an altered expression of osteoblast markers following SRC depletion or inhibition [13, 15, 44]. Here, we show that saracatinib treatment did not alter *Src*, *Alp or Runx2* expression levels. The latter is in agreement with the role of SRC as a modulator of RUNX2 activity rather than expression [47]. On the other hand, osterix (*Osx*), which has been implicated in the regulation of alpha-1 type 1 collagen (*COL1A1)* transcription [48], osteocalcin (*Ocn*), and *Col1a1* expression were decreased, although the latter did not reach significance.

The SFK LYN has been implicated in IL-6 driven proliferation of CD45+ MM cells [49] and has a high mRNA expression level in patient-derived MM cells, while other SFKs including *SRC* are expressed at low levels (Figure 1). We observed no direct effect of saracatinib at pharmacological relevant concentrations on the proliferation of murine and human MM cell lines, which display varying degrees of IL-6 dependency and CD45 expression [50–52]. In addition, we found no additive or synergistic effects of saracatinib when combined with bortezomib, lenalidomide or dexamethasone (Figure 4 and Supplementary. Figure S2). This is contrary to dasatinib, which had minimal single agent activity in a phase 2 trial [53], but displayed synergy with bortezomib, melphalan en thalidomide in preclinical studies [54]. *In vivo*, when given in a prophylactic (5TGM.1) or in a therapeutic (5T2MM) setting, saracatinib did not result in a decreased tumor burden. This contrasts with findings in breast cancer bone metastasis, where SRC is critical for the survival and outgrowth of malignant cells in the BM [55].

Next, we assessed the effect of saracatinib on the development of MM-associated osteolytic bone disease (Figure 5 and 6). In both the 5TGM.1 and the 5T2MM model we observed a marked prevention of bone loss after saracatinib treatment, with trabecular bone volume and number restored to levels observed in healthy mice. Trabecular thickness and separation were differentially affected during MM development in the 5TGM.1 and 5T2MM models, which likely reflects the different development rate of MM as well as the different dependency of MM cells on, and thus interactions with, the BM microenvironment in these models. This could also explain the difference in both the number and size of cortical lesions during MM development in both models. In the 5TGM.1 model, saracatinib treatment decreased the number and size of cortical perforations and partially restored cortical thickness. However, saracatinib did not have such an effect in the 5T2MM model while other therapeutic strategies, both directed at inhibiting osteoclast activity [56, 57] or at promoting osteoblast differentiation [58], did. This is unlikely due to the delay in the start of treatment of these mice, considering serum paraprotein levels start to increase at 7-8 weeks in the 5T2MM model after which osteolytic bone disease develops [59]. Alternatively, the treatment time could have been too short to observe effects on cortical bone given its remodeling rate is slower compared to trabecular bone. Finally, we performed histomorphometrical analyses (Figure 7). Saracatinib did not prevent the reduced bone formation observed in myeloma-bearing mice. Of note, the decreased collagen secretion following saracatinib treatment we observed *in vitro* did not result in a further decrease in osteoid surface *in vivo*. Our findings are in agreement with the model that reduced bone formation in MM mainly occurs through the inhibition of Wnt-signaling in osteoblasts by factors such as DKK-1 and sclerostin. Both osteoclast surface and numbers did not differ between naive healthy mice and saracatinib-treated myeloma-bearing mice. Although there is a definite trend towards a decrease in these 2 parameters in saracatinib- compared to vehicle-treated myeloma-bearing mice, this did not reach significance. It is possible that this is related to the high degree of variation in BM infiltration of MM cells, and thus osteoclastogenesis, between individual mice. In addition, this could be explained by a compensatory mechanism in the BM microenvironment that is not present in *in vitro* cultures. Similar to the phenotype observed in *Src*−/− mice [43], saracatinib could only block bone matrix resorption while osteoclast differentiation capacity is maintained in this more complex setting.

Although previous studies support a strong interdependency between MM cells and osteoclast activity [6, 60], we did not observe a decrease in tumor burden following saracatinib treatment in either model even though bone loss was prevented. This is in agreement with previous studies in which either a complete lack of, or an insignificant trend towards, a decrease in MM tumor burden was reported following successful treatment of bone disease, and this both in the 5TGM.1 [61, 62] and the 5T2MM models [56, 58]. Conversely, other studies do report anti-MM effects following successful treatment of bone disease [5, 63–65]. Thus, additional studies are needed to further elucidate the interplay between osteolytic bone disease and MM tumor growth and to distinguish between direct and indirect effects.

This study further establishes SRC inhibition as a promising approach for the treatment of MM-associated osteolytic bone disease. Furthermore, such a strategy might also be beneficial for the treatment of metastasis-induced bone disease. We show that saracatinib treatment results in a prevention of bone loss in 2 murine MM models. Additional clinical trials with SRC inhibition in cancer-induced bone disease are currently ongoing, including SarCaBon (clinicaltrials.gov: NCT02085603), a phase II trial to evaluate efficacy of saracatinib for the treatment of cancer-induced bone pain. Bone turnover will be assessed as a secondary outcome measure in this trial. Our study warrants direct evaluation of bone parameters and disease progression in MM patients treated with saracatinib or with novel compounds with a similar SRC kinase inhibitory profile, such as AZD0424 (clinicaltrials.gov: NCT01668550).

## MATERIALS AND METHODS

### Saracatinib

Saracatinib (AZD0530) was kindly provided by AstraZeneca (Macclesfield, UK). For in vivo studies, saracatinib was dissolved in a 0.5% hydroxypropylmethylcellulose solution containing 0.1% tween-80 (both from Sigma-Aldrich, St. Louis, USA). Mice received daily doses of 25 mg/kg saracatinib or vehicle solution by oral gavage. For *in vitro* studies, a stock solution was prepared at 10 mM in tissue-culture grade dimethylsulfoxide (Sigma-Aldrich).

### Cell lines culture

RAW264.7 and 5TGM.1GFP+ cells (kindly provided by dr. G Mundy, Vanderbilt University, Nashville, TN, USA) were cultured in DMEM (Lonza, Verviers, Belgium), supplemented with 10% fetal bovine serum (FBS)(Sigma-Aldrich), 2 mM L-glutamine (Lonza) and 1% penicillin/streptomycin (P/S)(Lonza). MC3T3-E1 cells were cultured in αMEM (Lonza) supplemented with 10% FBS and 1% P/S. JJN-3, LP-1, U266 and 5T33 [66] cells were cultured in RPMI-1640 (Lonza) supplemented with 10% FBS, 2 mM L-glutamine and 1% P/S. Cell culture was performed at standard conditions (37°C/5% CO_2_). Unless otherwise indicated all cell lines were purchased from ATCC (Molsheim, France).

### SRC family kinase expression analysis

GEO2R analysis of publically available microarray data was carried out to examine the expression of SFKs targets of saracatinib in MM patients and healthy controls (GSE6477) [67] and during primary murine osteoclast (GSE57468) [68] and primary human osteoblast (GSE28205) differentiation. Data acquisition and normalization methods in these datasets has been described previously [67, 68]. The data on SFKs mRNA expression in MM patients was supplemented with publically available data on protein expression in MM cell lines (www.proteinatlas.org) [69].

### In vitro osteoclast differentiation, TRAP staining and actin staining

Tibias and femurs from C57/KaLwRij mice were flushed, the cells subjected to ficoll separation (Sigma-Aldrich) and mononuclear cells were cultured for 3 days in αMEM/10% FBS/1% P/S supplemented with 100 ng/ml recombinant murine M-CSF (Prepotech, London, UK). The monocytes were re-seeded on day 4 at a density of 6500 cells/cm^2^ and 100 ng/ml recombinant murine sRANKL (Prepotech) was added to the culture medium to induce osteoclast differentiation. The medium was refreshed on day 7 and cultures were stopped on day 10. RAW264.7 cells were seeded at a density of 30,000 cells/cm^2^ in αMEM/10% FBS/2 mM L-glutamine/1% P/S and 30 ng/ml recombinant murine sRANKL (Prepotech, London, UK). On day 3, the differentiation medium was refreshed and on day 4 the cultures were stopped. At the end of osteoclast cultures, cells were fixed in 4% paraformaldehyde, lysed for RNA extraction or stained for TRAP activity using the Leukocyte Tartrate-Resistant Acid Phosphatase kit (Sigma-Aldrich) according to the supplier's protocol. Actin ring formation was assessed by staining cultures with phalloidin-FITC (Sigma-Aldrich) followed by analysis on a Nikon A1R confocal fluorescent microscope (Nikon Instruments Europe, Amsterdam, the Netherlands)

### Osteoclast resorption assay

To assess the resorptive capacity of osteoclasts 3000 primary monocytes or 10,000 RAW264.7 cells were seeded on Osteo Assay 96 well plates (Corning, New York, USA) in osteoclast differentiation medium. The medium was refreshed every 3 days. After 12 days, a Von Kossa staining was performed to visualize non-resorbed matrix (see later). The number of resorption pits and average pit size were quantified using ImageJ software (NIH, Bethesda, USA).

### *In vitro* osteoblast differentiation

Primary murine BM derived MSCs were isolated as described previously [70] and seeded at a density of 12,500 cells/cm^2^. To induce osteoblast differentiation, confluent MSCs were cultured in αMEM/10% FBS/2 mM L-glutamine/1% P/S supplemented with 50 μg/ml ascorbic acid (Sigma-Aldrich) and 2 mM β-glycerolphosphate (Sigma-Aldrich) for 28 days. The differentiation medium was refreshed every 3 days. MC3T3-E1 cells were seeded at a density of 30,000 cells/cm^2^ in αMEM/10% FBS/1% P/S. After the cells reached confluence, the medium was supplemented with 50 μg/ml ascorbic acid and 2 mM β -glycerolphosphate for 14 days to induce osteoblast differentiation. The differentiation medium was refreshed every 3 days. At the end of the osteoblast cultures, the cells were lysed for RNA extraction or bone matrix stainings were performed.

### Proliferation assay

Cell proliferation and viability was assessed with the cell proliferation kit I (Roche, Mannheim, Germany) according to the supplier's protocol. In short, cultures in 96 well plates were incubated with 10 μl MTT labeling reagent for 4 hours at 37°C. Next, 100 μl solubilization reagent was added to each well and incubated overnight at 37°C. The next day, absorbance was measured at 570 nm on a Wallac 1420 Victor2 microplate reader (Perkin Elmer, Waltham, USA).

### Osteoblast wound healing assay

To assess cell migration after treatment with saracatinib, MC3T3-E1 cells were seeded at a density of 35,000 cells/chamber in αMEM/10% FBS/1% P/S in silicone culture inserts (Ibidi, Martinsried, Germany). After 24 hours, the culture inserts were removed leaving a 500 μm gap in the MC3T3-E1 monolayer. Four phase-contrast pictures of the remaining gaps were taken over a period of 24 hours with a TiS microscope (Nikon, Tokyo, Japan). The surface of the gaps was measured using NIS-Elements software (Nikon) and migration rates were calculated using linear regression analysis and determination of the slope.

### RNA extraction, cDNA synthesis and real-time PCR

RNA was extracted using the RNeasy Mini kit (Qiagen, Venlo, the Netherlands) according to the supplier's protocol. Isolated RNA samples were subjected to DNaseI (Roche, Vilvoorde, Belgium) digestion prior to determination of the purity and concentration on a ND-1000 spectrophotometer (Thermo Scientific, Wilmington, USA). cDNA synthesis was performed on 100 ng RNA with random hexamer primers using the Transcriptor First Strand cDNA Synthesis Kit (Roche) according to the supplier's protocol. Real-time PCR (qPCR) was performed on a Lightcycler 480 instrument (Roche) using Kapa SYBR Fast qPCR master mix (Kapa Biosystems, Wilmington, USA) using 250 nmol/L of the appropriate primers (Supplementary Table S1). Gene expression was normalized to β-actin and β2-microglubulin expression. All primers were synthesized by Integrated DNA Technologies (Leuven, Belgium). The cycling conditions were as follows: 3 min at 95°C, 40 cycles of 10 s at 95°C, 30 s at 60°C and 1 s at 75°C followed by a melting curve analysis. Measurements were performed at least in triplicate. To compare expression levels between different conditions the ΔCt method was used.

### Von Kossa and Sirius red staining

Von Kossa staining was performed to detect mineralized matrix nodules in the osteoclast resorption assay and in osteoblast cultures. Cultures were decellularized with 20 mM NH_4_OH (Sigma-Aldrich) for 30 minutes at room temperature. After rinsing, plates were incubated with 5% AgNO_3_ (Sigma-Aldrich) solution for 1 hour, rinsed, and incubated with 1% pyrogallol (Sigma-Aldrich) solution for 10 minutes at room temperature in daylight. Collagenous matrix was visualized with Sirius red staining. After fixation in 70% ethanol, cultures were rinsed and stained with 0.1% direct red (Sigma-Aldrich) in picric acid (Sigma-Aldrich) solution for 1 hour at room temperature. Both Von Kossa and Sirius red staining were quantified with ImageJ software.

### The 5TGM.1 and 5T2MM murine multiple myeloma models

The 5TMM murine models originate from spontaneously developed MM in elderly C57BL/KalwRij mice [71–73]. The 5TGM.1GFP+ cell line was derived from 5T33MM cells [74]. The *in vivo* growing 5T2MM cells have been propagated by i.v. transfer of 2×10^6^ cells from diseased mice in younger syngeneic C57BL/KalwRij mice as described previously [72, 75]. C57BL/KalwRij mice were purchased from Harlan (Horst, the Netherlands). All animals had free access to food and tap water and they were housed and treated following conditions approved by the ethical committee for animal experiments of the University of Liege and the Vrije Universiteit Brussel (ULg license no. 1336 and VUB license no. 13-281-1). The 5TGM.1 model develops MM over the course of 30 days after i.v. injection of 2.5×10^5^ 5TGM.1GFP+ cells in 200 μl serum-free DMEM medium in C57BL/KalwRij mice. The induced MM disease is characterized by a selective BM infiltration and moderate osteolytic bone disease. BM infiltration of MM cells in these mice was determined by FACS detection of GFP+ cells on a FACSCalibur flow cytometer (BD Biosciences, Erembodegem, Belgium). MM develops within 10-12 weeks in the 5T2MM model and the disease in these mice is characterized by BM infiltration of 5T2MM cells and the occurrence of severe osteolytic lesions. In these mice, plasmacytosis was determined by staining cytosmears of the BM with May-Grunwald-Giemsa.

### Treatment of multiple myeloma-bearing mice with saracatinib

For the 5TGM.1 cohort, 14 male mice were inoculated with 5TGM.1GFP+ cells at 8 weeks old. Of the myeloma-bearing mice, 7 were given vehicle solution and 7 were given 25 mg/kg/day saracatinib by oral gavage starting at day one after injection. Six male mice were included as naive tumor-free controls. For the 5T2MM cohort, 22 female mice were injected at 8 weeks old and divided in a vehicle-treated myeloma-bearing group (n=12) and a saracatinib-treated myeloma-bearing group (n=10). In this cohort, 25 mg/kg/day saracatinib treatment was started at 5 weeks post-injection when a paraprotein could be detected by serum electrophoresis. Eight female mice were included as naive tumor-free controls. All mice were sex- and age-matched and they were sacrificed when the first mice of a cohort showed signs of established MM, i.e. at 4 weeks post-injection for the 5TGM.1 cohort and at 11 weeks post-injection for the 5T2MM cohort.

### Micro-computed tomography

One tibia and one femur were isolated after sacrifice of each mouse. The bones were fixed overnight in 2% paraformaldehyde/PBS solution at 4°C. The next day, the fixation solution was replaced by PBS and the bones were stored at 4°C. Micro-computed tomography (μCT) was performed on the distal femur and proximal tibia with a Skyscan 1172 (Bruker, Kontich, Belgium). The scanner source was set at 50 kV and 200 μA, and a 0.5 mm aluminum filter was applied. The pixel size was 5 μm^2^. Images were captured every 0.4 degrees through 180 degrees of rotation using Skyscan software (Bruker). Reconstruction was performed with NRecon software (Bruker). Morphometric 3D analysis of trabecular bone was performed on proximal tibias on a 1.5 mm section starting 0.5 mm from the growth plate using CTAnalyzer software (Bruker, version 1.14.4.1). Morphometric 2D and 3D analysis of cortical bone was performed in distal femurs on a 0.5 mm section starting 3 mm from the growth plate. 3D images of bones were generated using CTVol software (Bruker). The number of cortical perforations was counted manually and blinded on radiographs.

### Bone histomorphometry

Histomorphometric parameters were measured on femurs from mice in the 5TGM.1 cohort. Isolated femurs were dehydrated in ascending alcohol concentrations, defatted in xylene and embedded in methylmethacrylate. All histomorphometric parameters were recorded as recommended by the American Society for Bone and Mineral Research Histomorphometry Nomenclature Committee [76] and measured using the Bonolab software package designed for bone histomorphometry (Microvision, Evry, France). Osteoid surface and osteoblast surface were measured on sections stained with toluidine blue, aniline blue and Goldner's trichrome (Sigma-Aldrich). Osteoclasts were detected by tartrate-resistant acid phosphatase (TRAP) staining (Sigma-Aldrich). Briefly, sections were stained for acid phosphatase using naphthol ASTR phosphate as substrate in the presence of 50 mM tartrate with hexazotised pararosaline, and counterstained with methyl green. TRAP-positive cells were counted in the whole epiphysis (×25), and expressed as the number of osteoclasts per bone volume. In addition, osteoclast surface was determined.

### Statistical analysis

All *in vitro* experiments were performed in triplicate. Results are shown as means +/− standard error and representative pictures are shown. For comparisons of 2 means, a Student t-test was used. For comparisons of multiple means, a one-way ANOVA was used, followed by a Dunnett's post-hoc test (SFKs expression analysis and MTT assays) or Tukey's post-hoc test (other experiments). All statistical analyses were performed with Prism 5 software (Graphpad software, La Jolla, USA). P-values below 0.05 were considered significant and p-values are represented as follows: *: p<0.05, **: p<0.01, ***: p<0.001.

## SUPPLEMENTARY FIGURES AND TABLES


