# Supplementary material for: SRC kinase inhibition with saracatinib limits the development of osteolytic bone disease in multiple myeloma
Source: Oncotarget. 2016 Apr 15;7(21):30712–29. doi: 10.18632/oncotarget.8750 (PMC5058712; doi:10.18632/oncotarget.8750)
Supplement: Supplementary file 1 [file oncotarget-07-30712-s001.pdf]

SRC kinase inhibition with saracatinib limits the development of osteolytic bone disease in multiple myeloma

SUPPLEMENTARY TABLE AND FIGURES

Supplementary Table S1: Real-time PCR primer sets. Real-time PCR forward and reverse primers used to assess mRNA expression in murine osteoclast and osteoblast cultures.

Gene	Forward	Reverse
Src	AGAGGGAGACTGGTGGCT	ACAGAGAGGCAGTAGGCAC
Osteoclast function		
Nfatc1	TGAGGCTGGTCTTCCGAGTT	CGCTGGGAACACTCGATAGG
Ctsk	CAGCAGAGGTGTGTACTATG	GCGTTGTTCTTATTCCGAGC
Trap	TCCTGGCTCAAAAAGCAGTT	ACATAGCCCACACCGTTCTC
Dcstamp	TTGCCGCTGTGGACTATCTG	GAATGCAGCTCGGTTCAAAC
Mmp9	GTCCAGACCAAGGGTACAG	GGTATAGTGGGACACATAGTG
Osteoblast function		
Runx2	AAATGCCTCCGCTGTTATGAA	GCTCCGGCCCACAAATCT
Osx	AGCGACCACTTGAGCAAACAT	GCGGCTGATTGGCTTCTTCT
Col1a1	GCAACAGTCGCTTCACCTACA	CAATGTCCAAGGGAGCCACAT
Ocn	GCAATAAGGTAGTGAACAGACTCC	GTTTGTAGGCGGTCTTCAAGC
Alp	ATCTTTGGTCTGGCTCCCATG	TTTCCCGTTCACCGTCCAC
Reference genes		
β-act	TCTGGCTCCTAGCACCATG	AAAACGCAGCTCAGTAACAG
B2MG	GCTACGTAACACAGTTCCAC	TGATGCTTGATCACATGTCTCG

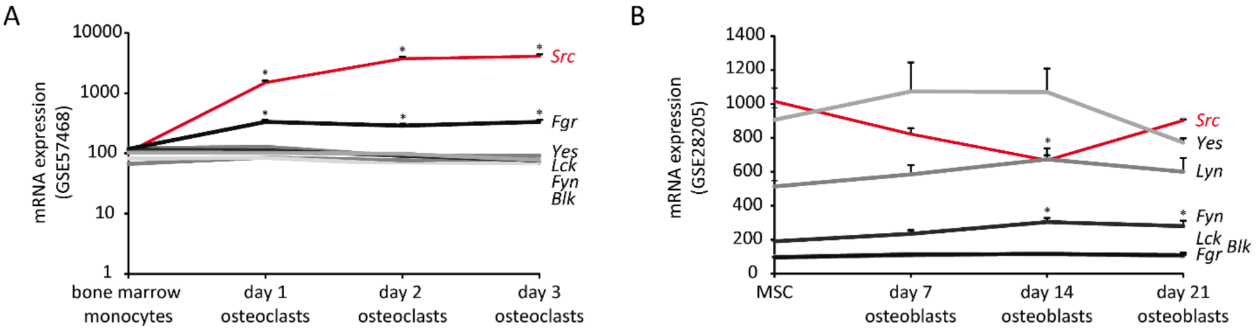

Supplementary Figure S1: SRC family kinase expression during osteoclast and osteoblast differentiation. A. SFK mRNA expression during primary murine osteoclast differentiation (GSE57468, n=2 experiments, *: p<0.05). **B.** SFK mRNA expression during primary human osteoblast differentiation (GSE28205, n=3 experiments, *: p<0.05). All data are represented as mean +/- standard error.

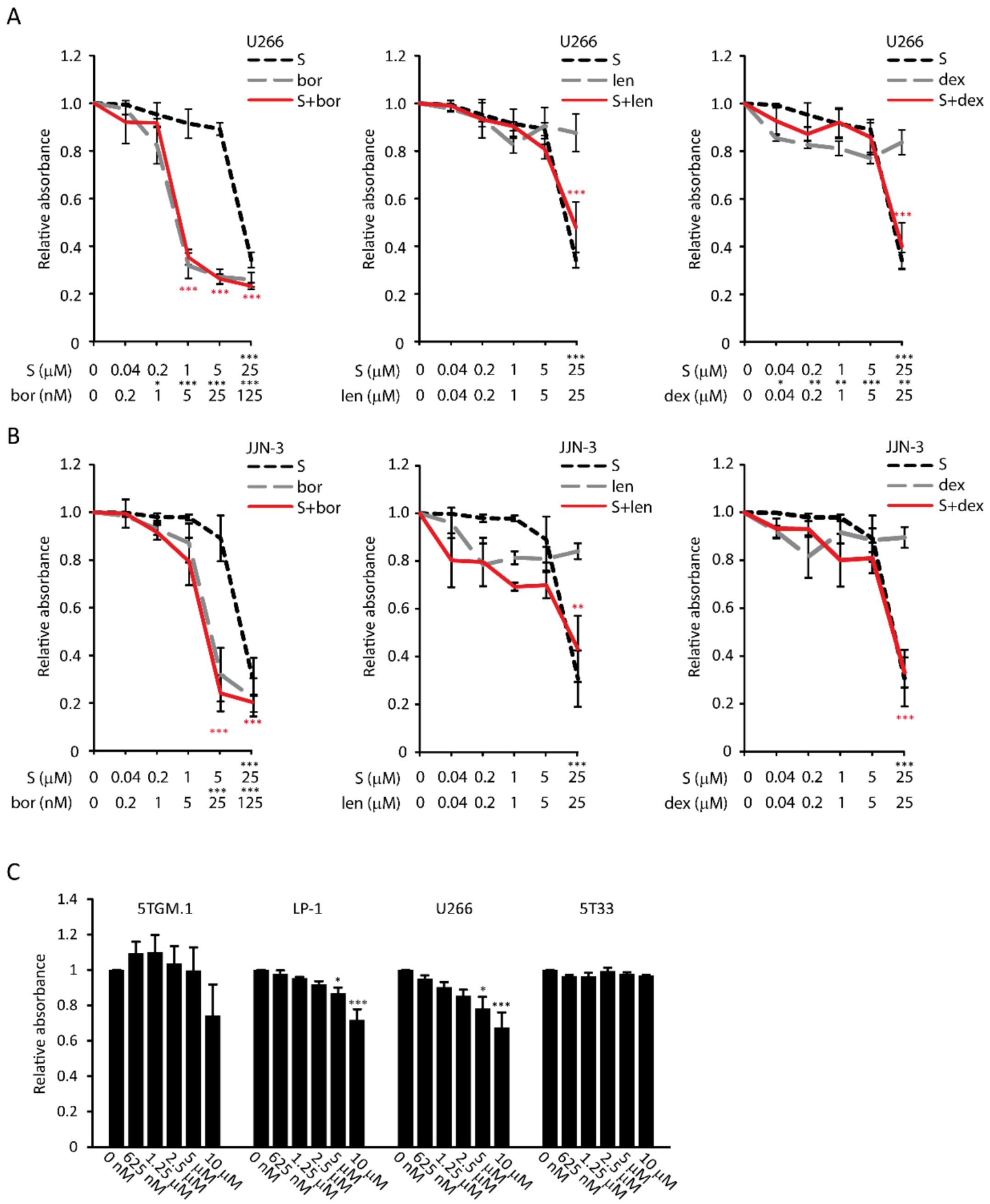

Supplementary Figure S2: Effect of saracatinib alone or in combination with standard drugs on multiple myeloma cell proliferation. A. MTT assays on human U266 myeloma cells treated with saracatinib (S), bortezomib (bor), lenalidomide (len), dexamethasone (dex) or combinations of saracatinib with these drugs. Drug concentrations and significance of single drug treatment compared to controls are noted on the X-axis. Significance of drug combinations compared to controls are noted in the figure (in red). No significant synergistic or additive effects were detected (n=3 experiments, *: p<.0.05, **: p<0.01, ***: p<0.001). **B.** MTT assays on human JJN-3 myeloma cells. **C.** MTT assays on murine 5TGM.1 and 5T33 cells and human LP-1 and U266 cells treated with saracatinib (n=4 experiments, 5T33: n=2 experiments, **: p<0.01, ***: p<0.001). All data are represented as mean +/- standard error.

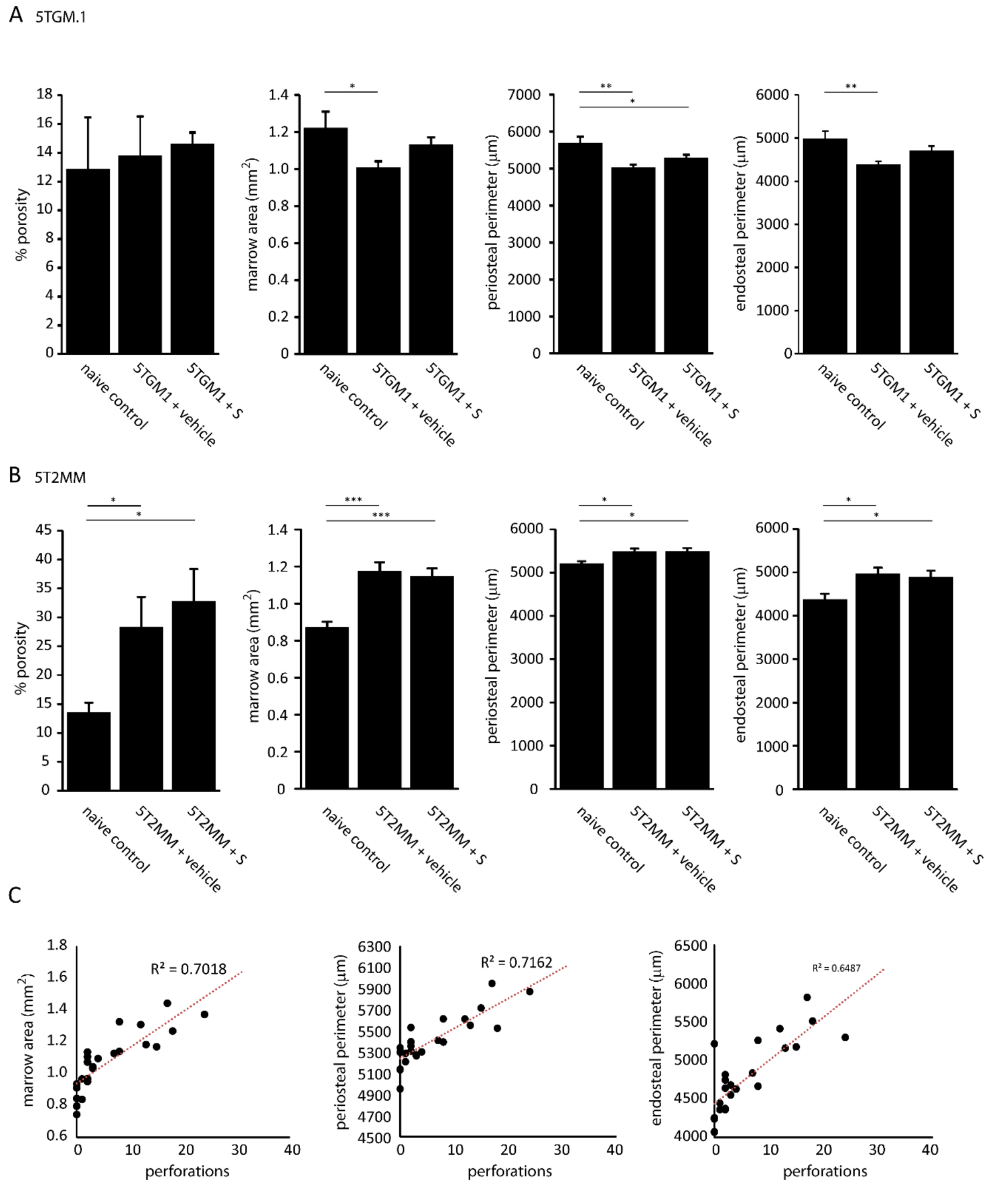

Supplementary Figure S3: Additional cortical parameters in the 5TGM.1 and 5T2MM models. A. Porosity, marrow area, periosteal and endosteal perimeter in naive control, 5TGM.1 myeloma-bearing vehicle-treated and 5TGM.1 myeloma-bearing saracatinib-treated mice (n=6/7/7 bones, *: p<.0.05, **: p<0.01). **B.** Porosity, marrow area, periosteal and endosteal perimeter in naive control, 5T2MM myeloma-bearing vehicle-treated and 5T2MM myeloma-bearing saracatinib-treated mice (n=8/12/10 bones, *: p<0.05, ***: p<0.001). **C.** Correlation of marrow area, periosteal and endosteal perimeter with the number of cortical perforation in the 5T2MM model. It should be noted that due to the sometimes very large lesions in the 5T2MM model, these data can be exaggerated (suboptimal gap closing algorithm for large gaps resulting in inflated marrow area and endosteal/periosteal perimeter). The number of lesions indeed correlates with marrow area, endosteal and periosteal perimeter in the 5T2MM model.
